# A Rare Double Primary Tumor of the Pancreas and the Esophagus

**DOI:** 10.7759/cureus.4955

**Published:** 2019-06-20

**Authors:** Jinendra Satiya, Mohit Girotra

**Affiliations:** 1 Internal Medicine, University of Miami, John F Kennedy Medical Center, Atlantis, USA; 2 Gastroenterology and Hepatology, University of Miami, Miller School of Medicine, Miami, USA

**Keywords:** double primary, pancreas cancer, esophageal cancer, endoscopic ultrasound, esophageal nodule, pancreas mass

## Abstract

Primary pancreatic cancer with synchronous primary tumors from other organs is extremely rare. Hereditary conditions, such as Lynch syndrome, Peutz-Jeghers syndrome, and hereditary pancreatitis, are linked to 10% of pancreatic cancers and may increase the risk of secondary malignancies from other organs. Only three cases of synchronous cancers of the pancreas and esophagus have been reported in the literature. Interestingly, patients with double primary tumors have been observed to have better overall survival than those with pancreatic cancers alone. We report the fourth such case of a 54-year-old male diagnosed with a double primary cancer of the pancreas and the esophagus diagnosed with endoscopic ultrasound (EUS).

## Introduction

Pancreatic ductal adenocarcinoma (PDAC) is the third leading cause of death from a solid malignancy in the United States, with a five-year overall survival rate as low as 8%. Pancreatic cancer with synchronous primary tumors is rare with a reported incidence of 0.75% to 20.0% [1-3], with common locations of the associated primary tumors being the stomach, colon, rectum, lung, and thyroid [4]. Hereditary conditions, such as Lynch syndrome, Peutz-Jeghers syndrome, and hereditary pancreatitis, are linked to 10% of pancreatic cancers and may increase the risk of secondary malignancies from other organs.

To the best of our knowledge, only three cases of synchronous cancers of the pancreas and the esophagus have been reported in the literature [5-7]. We report the fourth such case of a double primary of the pancreas and the esophagus, which was interestingly diagnosed with endoscopic ultrasound (EUS).

## Case presentation

**Figure 1:**
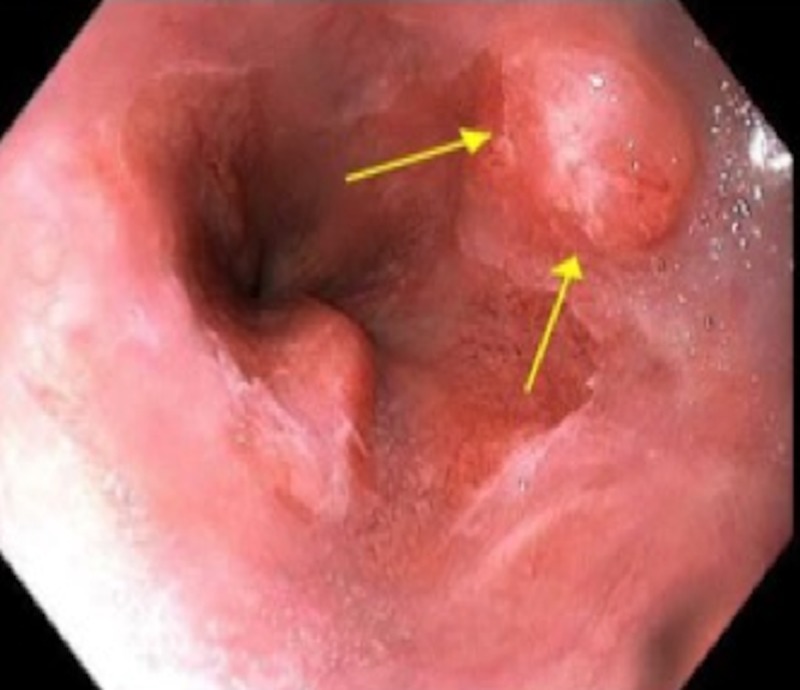
An upper endoscopy demonstrating a nodule at the gastroesophageal junction (GEJ)

A 54-year-old man with a history of hypertension and smoking presented with nine months of epigastric pain, decreased appetite, and a 30 lb. weight loss. His family history was significant for PDAC in maternal aunt and breast cancer in another aunt. Abdominal computed tomography (CT) performed at an outside facility showed a mass in the pancreas, measuring 4 cm x 3.8 cm x 2.6 cm, encasing the superior mesenteric artery, and he was referred to our institution for further management. His hepatic panel showed an elevated alanine transaminase at 160 IU/L (normal: 7 - 56 IU/L) and an alkaline phosphatase of 256 IU/L (normal: 44 - 147 IU/L). Carbohydrate antigen 19-9 (CA 19-9) was elevated at 177 U/mL (normal: 0-37 U/mL). An esophagogastroduodenoscopy (EGD) demonstrated a nodule at the gastroesophageal junction (Figure 1). 

**Figure 2:**
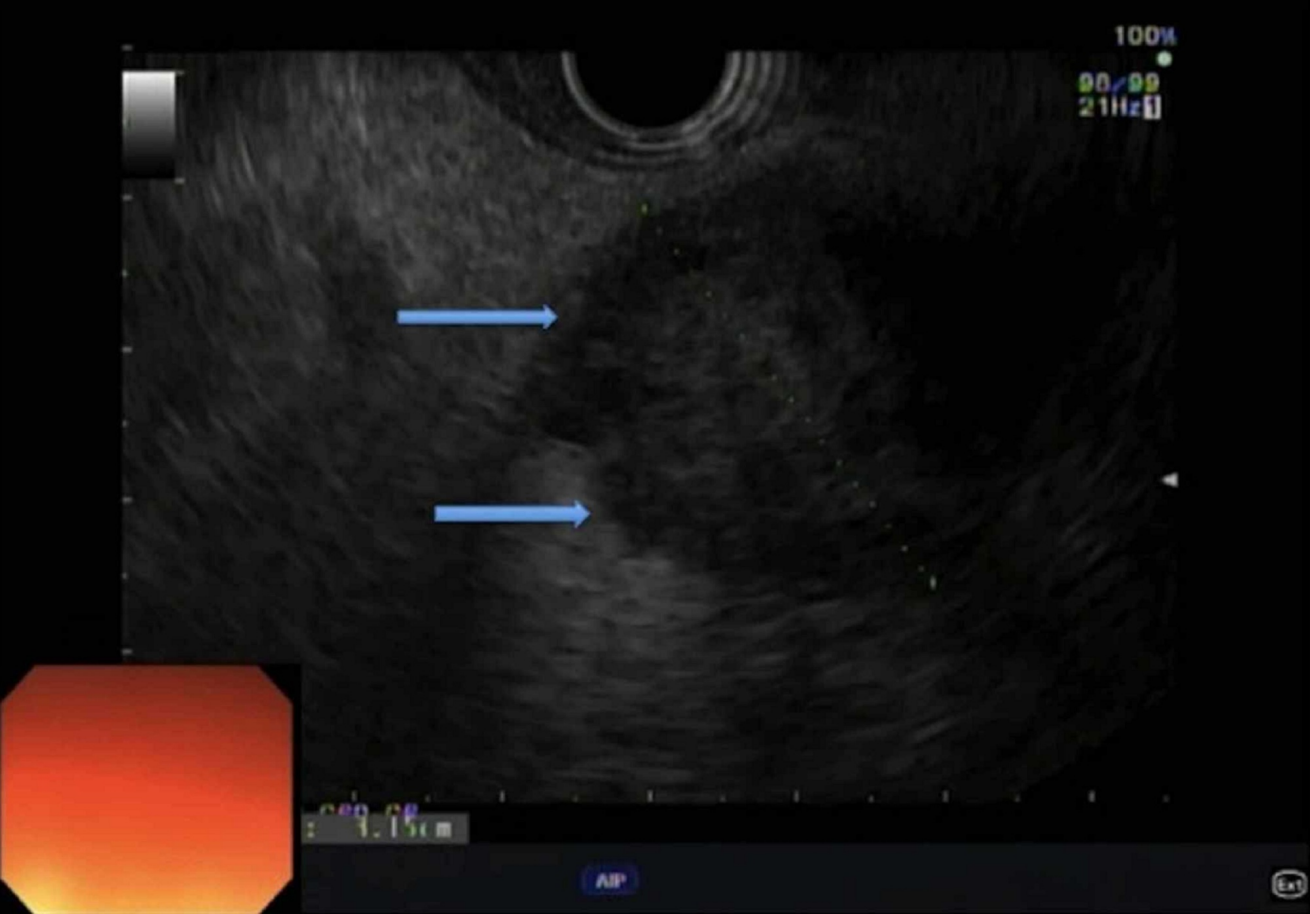
An upper endoscopic ultrasound demonstrating a hypoechogenic area between the head and body of the pancreas

An endoscopic ultrasound (EUS) revealed an ill-defined, hypoechogenic, lobular area, measuring 19.6 mm x 24.7 mm, located between the head and body of the pancreas (Figure 2). 

**Figure 3:**
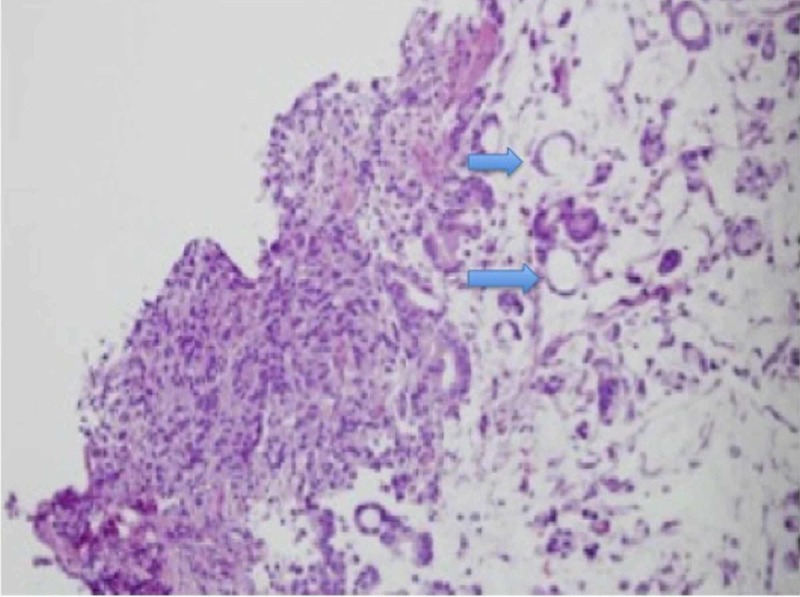
Distal esophageal nodule biopsy revealing esophageal adenocarcinoma with mucinous features (blue arrows)

No lymph nodes were seen. The patient was additionally found to have an approximately 1 cm nodule at the gastroesophageal junction (GEJ), which was limited to the mucosa and submucosa, and removed with endoscopic mucosal resection (EMR). The pathology from the pancreatic mass confirmed adenocarcinoma (positive for CK7, CK20, DPP4, and CDX2) with microsatellite stability (with an intact expression of MLH1, MSH2, MSH6, and PMS2). The pathology of the GEJ nodule proved to be adenocarcinoma with mucinous features (microsatellite stable but with KRAS amplification) with NOTCH1 E905* and TP53 C135Y genomic alterations (Figure 3).

The patient was diagnosed with a double primary of the pancreas and the esophagus and started on FOLFIRINOX (5-fluorouracil, leucovorin, irinotecan, and oxaliplatin) in consultation with medical oncology. He has received 10 cycles of FOLFIRONOX thus far and was doing well at his six-month follow-up.

## Discussion

The increase in the incidence of multiple primary malignancies can be attributed to an increase in the longevity of patients, the advent of diagnostic tools with higher sensitivity, and newer modalities of treatment. Despite recent advances in the field of oncology, PDAC remains a rapidly fatal disease. Although definite causes of PDAC are yet to be identified, there are known risk factors precluding to a higher probability, such as chronic pancreatitis, increasing age, obesity, smoking, race, family history, genetic predisposition, and environmental factors [8]. 

Multiple primary malignancies of solid organs are rare. Metastatic lesions should be excluded before a diagnosis of multiple primary cancers can be made. The tumors must be histologically different and must involve different organs. They can be categorized as metachronous when tumors follow one another and synchronous when they arise simultaneously or within six months from the primary malignant tumor [9]. Metachronous are more frequent than synchronous tumors with a ratio of 2.7:1 [10].

PDAC is linked to a high incidence of other malignancies of the gastrointestinal tract [11]. Factors such as chronic inflammation, dietary factors, co-morbidities, extensive smoking history, and genetics could potentially play a causative role in the occurrence of synchronous malignancies, with studies identifying microsatellite instability more frequently in this patient population [12]. Despite the known occurrence of synchronous malignancies, metastases from PDAC still remain much more common, with the commonest site being liver, followed by the peritoneum and then the lung. 

In a series of 113 patients with PDAC and double primary tumors, 44 patients (38.9%) were identified as having synchronous double primary tumors and 69 (61.1%) were diagnosed with metachronous double primary tumors [7]. The stomach was the most common location for a double primary with 26 patients, accounting for 21.5% of the 113 patients. The colon and rectum were the second-most common sites with 25 patients (20.7%), followed by the lung with 18 patients (14.9%), thyroid (13 patients), and liver (nine patients). A Japanese cohort study of 337 resected lung cancer cases over seven years identified 24 cases of multiple primaries, out of which only two cases were of primary lung and pancreas [13]. 

The prognosis of patients with PDAC and a double primary cancer heavily depends on the prognosis of the pancreatic malignancy. Those that had their PDAC resected before the diagnosis of metachronous double primary tumors had better overall survival (OS) than patients whose PDAC was resected after the diagnosis of metachronous double primary tumors. These patients also had a higher overall survival (OS) compared to those with resected PDAC and synchronous double primary tumors. Patients who were diagnosed with PDAC before the metachronous double primary tumors have also been shown to have better survival [7]. Interestingly, pancreatic cancer patients with stomach, thyroid, and lung cancers have had better odds of survival ratios than those with pancreatic cancer only.

## Conclusions

As the survival associated with PDAC is poor, a second primary cancer is rarely seen in this patient population. Recent advances in diagnosis and treatment have led to an increased incidence of second primary cancers. The most common location for a second primary in a patient with PDAC is the stomach, followed by the colon and rectum, and lung. These patients are often found to have a better OS than those with only pancreatic cancer. Early diagnosis of these multiple primaries are key to improved patient outcomes. Our case is unique since this is only the fourth case of synchronous double primary malignancy of the pancreas and the esophagus described in the literature, and EUS played a major role in its detection. More research is required to identify associations between pancreatic and other solid organ malignancies.
